# Environmental benefits and concerns on safety: communicating latest results on nanotechnology safety research—the project DaNa^2.0^

**DOI:** 10.1007/s11356-016-6217-0

**Published:** 2016-02-23

**Authors:** D. Kühnel, C. Marquardt, K. Nau, H. F. Krug, F. Paul, C. Steinbach

**Affiliations:** 1 Department of Bioanalytical Ecotoxicology, Helmholtz Centre for Environmental Research - UFZ, Leipzig, Germany; 2 Institute for Applied Computer Sciences (IAI), Karlsruhe Institute of Technology (KIT), Eggenstein-Leopoldshafen, Germany; 3 International Research Cooperations, Empa - Swiss Federal Laboratories for Materials Science and Technology, St. Gallen, Switzerland; 4 Society for Chemical Engineering and Biotechnology (DECHEMA), Frankfurt am Main, Germany

**Keywords:** Nanomaterials, Nanoobjects, Nano ecotoxicity, Science communication, Knowledge base, Knowledge dissemination, Environmental impact

## Abstract

The use of nanotechnology and advanced materials promises to revolutionise many areas of technology and improve our daily life. In that respect, many positive effects on the environment are expected, either directly, by developing new technologies for remediation, filtering techniques or energy generation, or indirectly, by e.g. saving resources due to lower consumption of raw materials, or lower energy and fuel consumption due to reduced weight of vehicles. However, such beneficial effects of new technologies are often confronted by concerns regarding the safety of novel substances or materials. During the past 10 years, great effort has been put into research on potential hazards of nanomaterials towards environmental organisms. As the methodology for reliable assessment of nanomaterials was immature, many studies reporting contradictory results have been published, hindering both risk assessment for nanomaterials, as well as the knowledge communication to all involved stakeholders. Thus, DaNa^2.0^ serves as a platform to implement trusted knowledge on nanomaterials for an objective discussion.

## Introduction

Assessing the impact of new technologies or newly developed substances on humans and our environment is a challenge, even more so, when the applied test methods—both toxicological and analytical—are found to be partly inadequate and need amendments as it is in the case of nanotechnology (Krug 2014; Potthoff et al. 2015; Warheit and Donner 2015). This is illustrated by numerous publications in the field of nano-ecotoxicology, which although they have been investigating the impact of a number of nanomaterials on several organisms, almost never allow for explicit statements on potential hazards. This fact not only hampers the knowledge communication to all non-scientists (e.g. consumers), but it also complicates the transfer of the obtained results for other scientists.

Hence, reliable and understandable information on nanomaterials and nanotechnology is often missed. To bridge this gap, a web-based knowledge base (www.nanopartikel.info/en) has been developed. In an interdisciplinary approach, scientists of the DaNa^2.0^ expert team provide a knowledge base for more transparency wrapping up the results of current research on nanomaterials regarding their influence on humans and the environment in an understandable way. The selection of nanomaterials for inclusion into the knowledge base is driven by market-relevance as well as by applications. This is demonstrated on several nanomaterials applied for environmental purposes, some of which are elucidated in more detail in this article.

Powered by the need to provide clear information on risk and benefits of nanotechnology, the presentation of complex scientific data via the DaNa webpage www.nanopartikel.info/en addresses not only the scientific community but is just as much intended for the broader public, e.g. consumers, journalists, students or scientists from other research areas than nanotechnology. The collected knowledge integrated into the DaNa database could also contribute to the prioritisation of further research needs.

## Examples for applications of nanoproducts in an environmental context

### Water remediation (ground, surface and waste water)

In many areas with high industrial activity, ground and surface water is contaminated with organic pollutants such as perchloroethylene (PCE) and solvents such as benzene. The clean-up of those waters is laborious and cost-intensive, as conventional treatment methods rely on removing the contaminated water from the environment, destroying the pollutants, and then leading back the cleaned water to the environment. Nanomaterials open up the opportunity to directly introduce the clean-up agent into the contaminated water where the pollutants are then degraded on the spot (in situ) in a short time period. This is mainly due to the large specific surface area of the nanomaterials, which renders them very reactive. Some nanomaterials are very effective catalysts with a large potential to degrade pollutants, making the technology more cost effective than the conventional pump-and-treat methods. Current examples for environmental applications are iron-based nanomaterials such as zero-valent iron nanoparticles (Köber et al. 2014) or the nanocomposite Carbo-Iron® (Mackenzie et al. 2012). However, as an intentional release of nanomaterials into the environment takes place with the nanomaterials remaining in the environment after usage, concern on adverse effects on organisms was expressed. In consequence, potential adverse effects on biota need to be assessed in parallel to the development of novel remediation techniques (e.g. Weil et al. 2015).

Another area of applications for nanomaterials is the clean-up of contaminated industrial wastewater, which is not suitable for conventional treatment in wastewater treatment plants (Hildebrand et al. 2009; Hildebrand et al. 2008). Here, however, the clean-up technique is designed to separate the nanomaterials from the purified water before releasing it into the environment, hence significantly reducing the concern for detrimental effects on the environment.

### Zeolite nanomaterials in fertiliser and in water treatment

Zeolites are a class of highly porous material with channels and pores of nanometre diameters. These channels and pores are intended to store plant nutrients such as nitrogen and potassium and release them gradually. The benefit is that the fertiliser is used more efficiently because the slow release prevents run-off of nutrients during rain events, thereby decreasing over-fertilisation. However, to date, the application of nutrient-loaded zeolite fertiliser is constrained by high costs for zeolite purchase and loading (Liu and Lal 2015). Furthermore, zeolites are also applied for water cleaning purposes due to their high capacity to selectively adsorb ions, specifically metal cations such as Cu, Cr and Cd. In terms of environmental impact, zeolites are not of major concern as they are considered to be insoluble and their main constituents, aluminium and silicon, are considered non-toxic (Fruijtier-Pölloth 2009). In addition, naturally occurring zeolites were shown to reduce the bioavailability of toxic cadmium for fish (Ghiasi 2011).

### Nanopesticides

Pesticides or plant protection products are intended to protect crops from damage caused by insects, microorganisms or vermin such as nematods. The application of nanomaterials in this sector is also based on a number of expected benefits, including increased efficacy of the pesticide, while at the same time reducing the amounts of active ingredients. Likewise, the durability is increased, as some formulations allow a controlled gradual release of the pesticide, reducing the untargeted release into the environment and preventing detrimental effects on non-target species. Nanopesticides can contain a number of pristine engineered nanoparticles, such as metals, metal oxides, and nanoclays. Several formulation types have been suggested, such as emulsions and nanocapsules (Kah et al. 2013; Kah and Hofmann 2014; Kookana et al. 2014). For this type of application, benefits and risks have to be weighted carefully, but to date little research has been performed in the field of environmental fate and impact of nanopesticides.

As these examples demonstrate, there are many promising applications for nanomaterials in the environmental sector, which hold several advantages compared to conventional techniques or substances. At the same time, the necessity of an extensive assessment of the safety of novel technologies is evident. However, currently, the research into the risks of nanotechnology is lacking appropriate instruments to assess adverse effects towards humans and the environment in a reliable manner (Jemec et al. 2016; Kühnel and Nickel 2014). For this reason, the DaNa^2.0^ project aims at providing reliable information on nanomaterials suitable for a broad audience (Krug et al. 2014; Marquardt et al. 2013; Steinbach et al. 2012).

### The DaNa^2.0^ knowledge base

**Fig. 1:**
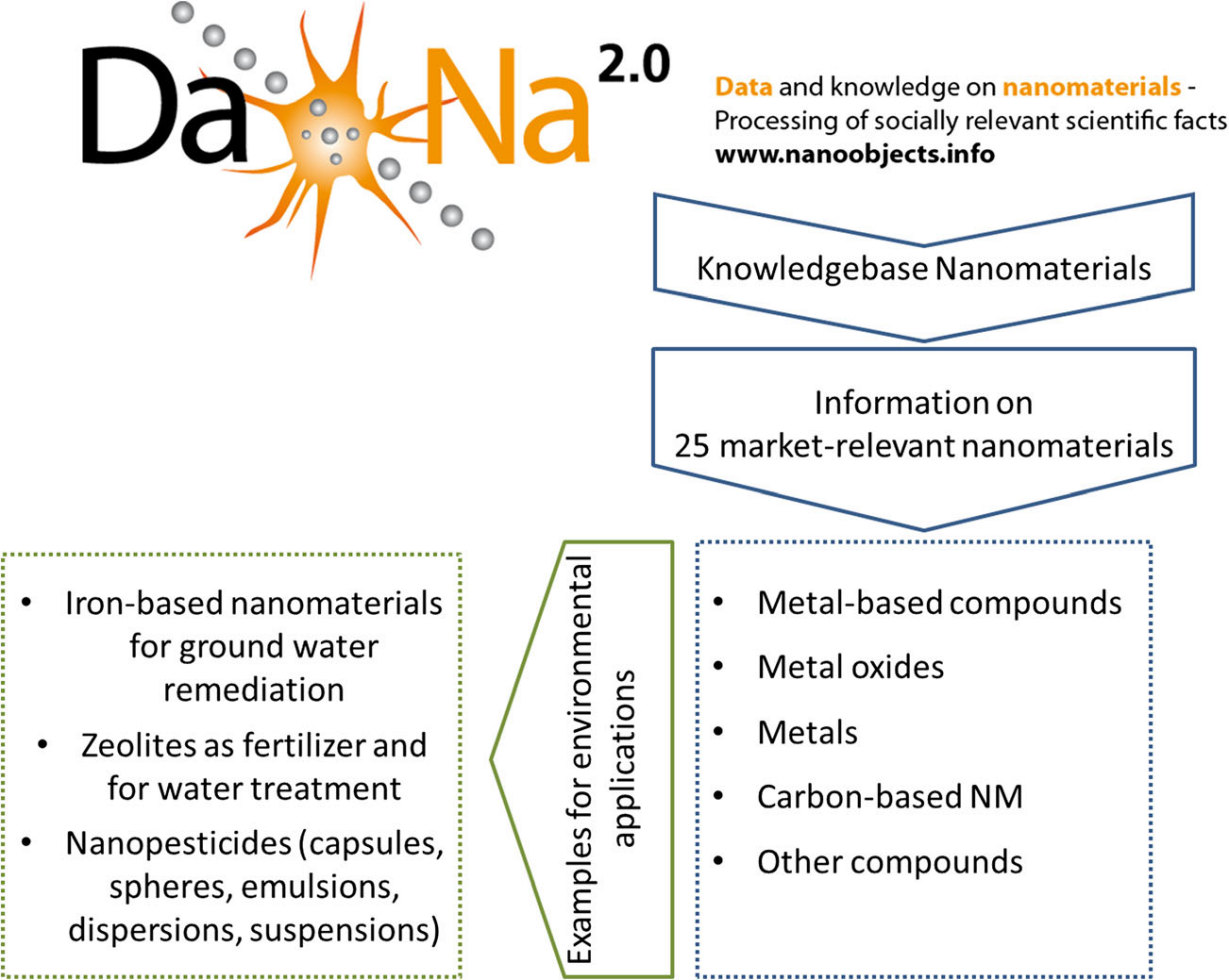
Overview on groups of nanomaterials contained in the DaNa^2.0^ knowledge base and examples with environmental relevance

The DaNa^2.0^ (*Data and Knowledge on nanomaterials – Processing of socially relevant scientific facts*) project team is evaluating research findings from the field of human and environmental nanotoxicology and presenting them together with material properties and applications for interested laymen and stakeholders. The core section of the DaNa^2.0^ web platform (www.nanopartikel.info/en) is a knowledge database, which provides a wealth of facts and data for engineered nanomaterials with regard to actual applications and their respective effects on humans and the environment (Fig. 1). The content within the knowledge base is derived from scientific literature and from various reports by an international and interdisciplinary expert team making use of a structured, criteria-based evaluation of the published literature (Kühnel et al. 2014; Marquardt et al. 2013).

**Fig. 2:**
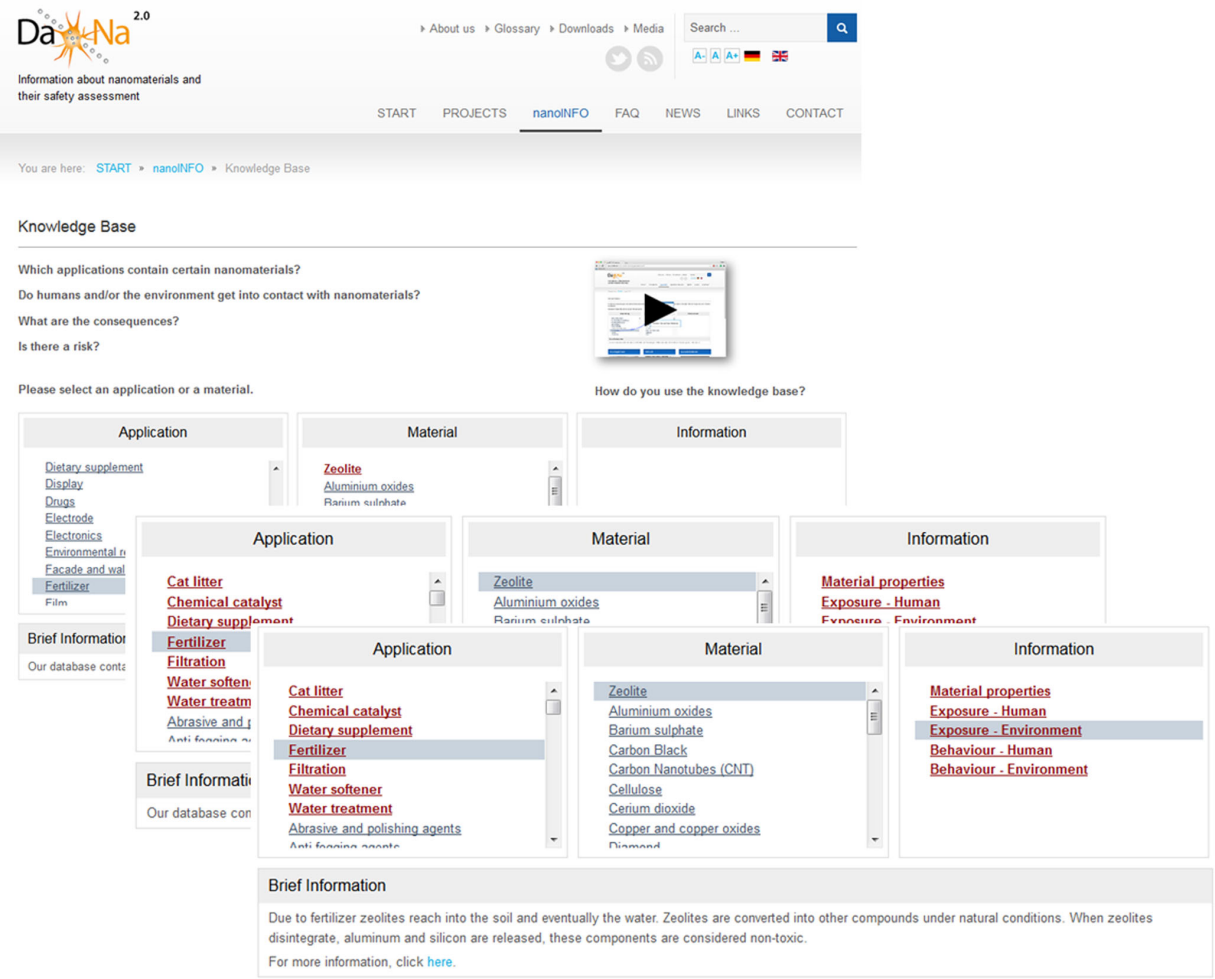
Access to information on nanomaterial safety according to application. By choosing ‘fertiliser’ in the application column, the material column highlights ‘zeolite’. Upon choosing zeolite, the information column provides access to in-brief information on material properties, exposure of man and environment, as well as uptake and behaviour (not available in this example). From there, more in-depth information is accessible. Moreover, the application column highlights now additional uses of this nanomaterial. (http://www.nanopartikel.info/en/nanoinfo/knowledge-base#anwendung=anwendung/60&material=material/28, Dec. 2015)

Currently, information on 25 market-relevant nanomaterials is provided, covering information on the material, more than 100 applications, as well as the toxicological aspects of the materials. The most important and user-friendly feature is the linkage of a specific nanomaterial to its applications or products. As illustrated in Fig. 2, the visitor of the web platform can first select an application of interest, and in combination with a certain nanomaterial used for this application is then guided to brief and more in-depth background information on the respective nanomaterials and related safety aspects.

This allows to access information in a graded way, according to background and interest of the recipients on specific issues. To achieve this, the content of the articles within the knowledge base is presented in four graded levels of complexity (Table 1).

**Table 1:** Graded levels of complexity realised in the DaNa^2.0^ knowledge base to address different backgrounds of our visitors

Level of complexity	Content	Anticipated recipient
One pager	Short and basic summary, covering the most striking information for each nanomaterial	Interested citizens/consumers (public-oriented)
Short summary	Subdivided into sections with regard to material information, exposure, uptake and behaviour of a specific nanomaterial	Interested citizens/consumers (public-oriented)
Detailed article	In-depth information for each section and nanomaterial	Journalists, stakeholders, scientists from other fields, regulators
Original references	List of the literature used to compile the detailed articles	Scientists from related fields, regulators

### Additional features of the DaNa^2.0^ web platform and the DaNa^2.0^ team

The DaNa^2.0^ knowledge base is encircled by various sections on the web platform, which provide further information about nanomaterials. This can be either more general, overarching information, such as in the basics section providing fundamental introductions into nanotechnology and safety issues. The cross-cutting section deals with overarching issues with significance to ENMs in general, for example on nanomaterials used in paints or toner as well as the relevance of nanoparticles for the human immune system. On the other hand, exploratory information is provided in the glossary and the FAQ section. The sections on research projects related to nanomaterials give an insight into the scientific landscape with a focus on Germany.

Based on the principles for careful scientific practice, the DaNa^2.0^ project team has also compiled a *Standard Operation Procedures* (*SOP*) *template* to fill in, as well-designed SOPs should be the basis for future research on the safety of engineered nanomaterials. Also, SOPs developed within research projects related to nanotechnology are published on the website, for example those describing the preparation of Carbo-Iron® and zeolite suspensions for ecotoxicological testing. By this, reliable SOPs are made available to researchers worldwide to foster harmonisation in nanomaterial testing.

**Fig. 3:**
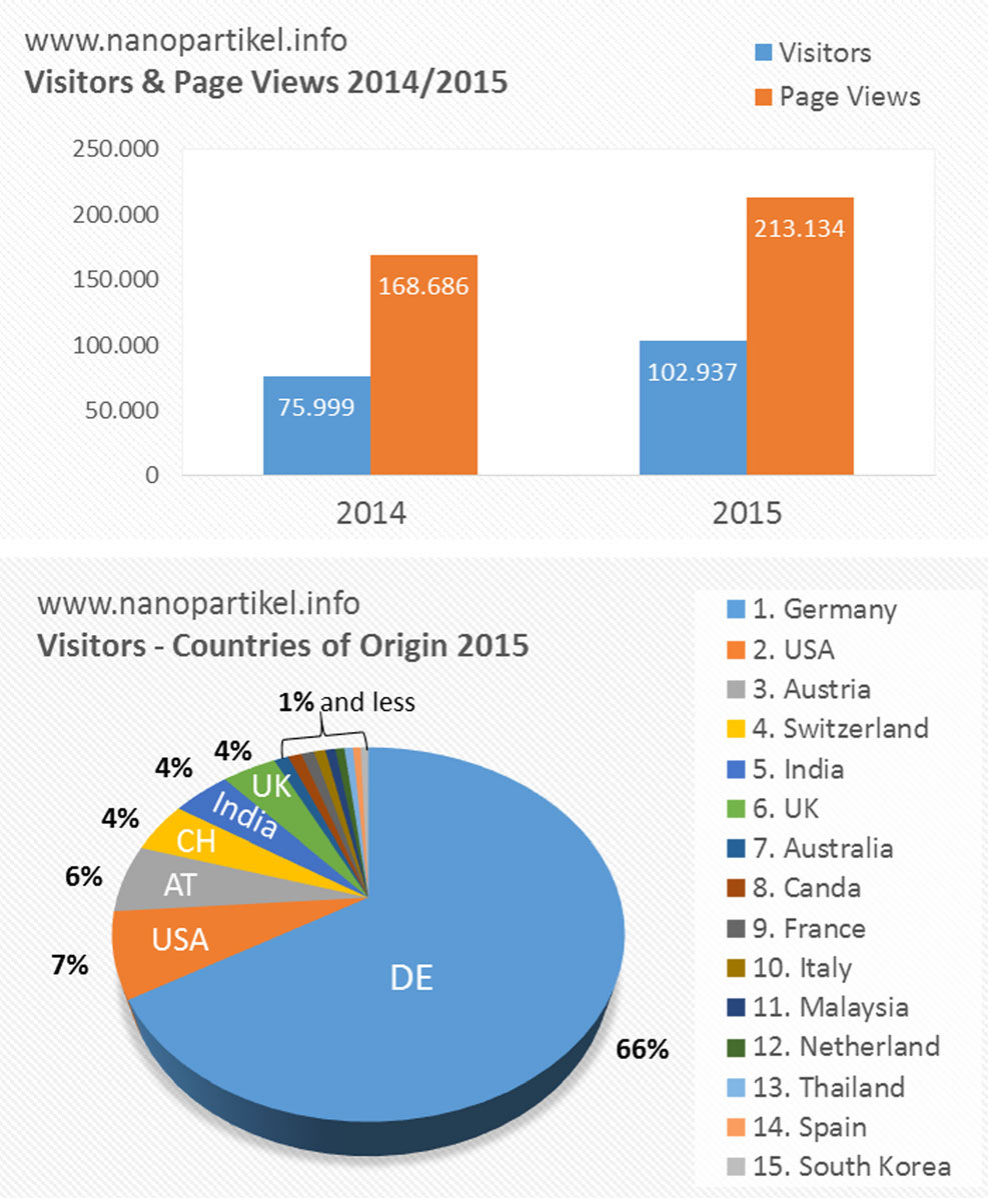
Access statistics for the DaNa2.0 website. In the *upper panel*, the number of visitors and the respective page view for the years 2014 and 2015 are depicted. The *lower panel* shows the distribution of visitors according to countries of origin

In addition to this, the scientists behind the DaNa^2.0^ web platform like to interact with people interested in ‘nano’ directly. The main aim of the teams’ dissemination activities is to provide the general public with sound and up-to-date information related to nanotechnology. Hence, we are actively participating in various dialogue processes such as citizen dialogues, discussions, fairs and conferences, and are in contact with other European information platforms and provide the opportunity to directly address questions to our experts via E-mail or twitter (@nano_info). These activities are appreciated worldwide which is reflected in the number of visitors on our website. These numbers as well as page views increase constantly since the launch of the website in 2009, reaching more than 100,000 visitors in 2015 (Fig. 3), viewing for more than 200,000 pages. Due to the international networking of the DaNa2.0 team, the page is used by visitors from all continents, with most visitors coming from Europe.

### Summary

Nanotechnology holds many promising applications for the environmental sector, but some of them are associated to concerns on detrimental effects towards the environment and environmental organisms. To provide reliable and unbiased information to several groups of recipients, the DaNa2.0 team set up the website www.nanopartikel.info/en. On this platform, latest research results related to nanotechnology and nanosafety are communicated in a structured and understandable way.
